# Spatial structuring of root-associated bacteria and metabolic landscapes in an endangered cliffside conifer *Thuja sutchuenensis* Franch

**DOI:** 10.1093/ismeco/ycag115

**Published:** 2026-04-23

**Authors:** Youwei Zuo, Yang Peng, Wenqiao Li, Zhijang Yang, Hongping Deng

**Affiliations:** Key Laboratory of Eco-Environment in the Three Gorges Reservoir Region, Ministry of Education, School of Life Sciences, Southwest University, Beibei, Chongqing 400715, China; Key Laboratory of Eco-Environment in the Three Gorges Reservoir Region, Ministry of Education, School of Life Sciences, Southwest University, Beibei, Chongqing 400715, China; Key Laboratory of Eco-Environment in the Three Gorges Reservoir Region, Ministry of Education, School of Life Sciences, Southwest University, Beibei, Chongqing 400715, China; Key Laboratory of Eco-Environment in the Three Gorges Reservoir Region, Ministry of Education, School of Life Sciences, Southwest University, Beibei, Chongqing 400715, China; Key Laboratory of Eco-Environment in the Three Gorges Reservoir Region, Ministry of Education, School of Life Sciences, Southwest University, Beibei, Chongqing 400715, China

**Keywords:** *T. sutchuenensis*, multi-omics, endangered plant, rhizosphere

## Abstract

Understanding how plant–microbe–soil interactions vary across spatial scales is essential for elucidating belowground ecological dynamics, particularly in rare and ecologically sensitive species. Here, we investigated the diversity, function, and assembly of root-associated bacterial communities in *Thuja sutchuenensis* Franch. along continuous vertical gradients. Through an integrative multi-omics approach, morphological assessment, and soil physicochemical profiling, we uncovered a compartmentalized and spatially structured rhizosphere system. We observed a marked enrichment of soil nutrients, particularly carbon, nitrogen, and available phosphorus, in the rhizosphere compared with bulk soil. Bacterial diversity declined in the endosphere, accompanied by taxonomic and functional shifts, including enrichment of Proteobacteria and Actinobacteriota and metabolic pathways related to host interaction and xenobiotic degradation. Root and soil metabolomes also showed depth-specific signatures, with surface compartments enriched in flavonoids and defense compounds, and deeper layers characterized by core metabolic functions. Co-occurrence network and correlation analyses highlighted key bacterial hubs and revealed strong links between nutrient levels and bacterial diversity. Together, this study provides new insight into the organization of plant–soil ecosystems and offers a framework for conservation and ecological restoration strategies for endangered cliffside species.

## Introduction

*Thuja sutchuenensis* Franch., commonly known as Sichuan thuja, is an endangered and rare conifer species endemic to China, primarily distributed along steep cliffs and canyon forests in the Daba Mountains at elevations between 1000 and 2100 m [1, 2]. In its native habitat, *T. sutchuenensis* develops a shallow but laterally extensive root system that anchors into rocky substrates. The species typically grows in well-drained, acidic mountain yellow soils with low organic matter and high microhabitat heterogeneity [1, 2]. As a Tertiary relict species that survived Quaternary glaciations, *T. sutchuenensis* holds exceptional value for studies in genetics, systematics, and paleoecology, and is often referred to as a “living fossil” [2, 3]. Ecologically, it serves as a dominant tree species in its native habitat, contributing to soil stabilization on rocky slopes, preventing erosion, and supporting local biodiversity by offering unique microhabitats [4, 5]. However, due to increasing anthropogenic disturbances and habitat fragmentation, the population of *T. sutchuenensis* has declined significantly, placing its associated ecosystems at risk of degradation [6]. Investigating its root-associated bacterial communities at spatial scales can offer critical insights into the mechanisms underlying its environmental adaptability and provide valuable knowledge for the conservation and restoration of this ecologically significant species.

Root-associated microbial communities are fundamental components of the plant holobiont, the host plant together with its associated microbiota, which collectively function as an ecological unit and influence plant development, nutrient acquisition, and environmental interactions [7, 8]. In particular, bacterial communities represent a dominant and functionally diverse component of the root-associated microbiome. These communities include diverse rhizobacteria and endophytic bacteria that establish complex associations with host plants across the soil–root continuum [9, 10]. For example, many bacterial taxa contribute to nutrient cycling through processes such as nitrogen transformation and organic matter decomposition, while others modulate plant responses via phytohormone production or signaling interactions. In environmentally constrained systems, such as nutrient-poor or drought-prone habitats, bacterial communities can play important roles in mediating resource availability and influencing plant–soil interactions [11, 12]. In addition, increasing evidence shows that bacterial communities are highly structured across spatial niches, including bulk soil, rhizosphere, root surface (episphere), and endosphere, reflecting strong environmental filtering and host selection along the soil–root interface [13, 14]. Despite these advances, how bacterial diversity, functional potential, and activity vary simultaneously across vertical soil gradients and root-associated compartments remains poorly understood, particularly in spatially heterogeneous habitats such as those occupied by *T. sutchuenensis*.

Spatial heterogeneity is a defining feature of mountainous ecosystems and plays a critical role in shaping bacterial community structure and function. Soil properties such as moisture, nutrient availability, and pH can vary over centimeter-scale distances, leading to strong spatial gradients in bacterial composition and activity [15–17]. Traditional sampling approaches often overlook this micro-scale variability, limiting our ability to resolve localized ecological processes. In contrast, integrating spatial sampling with multi-omics approaches provides an opportunity to link bacterial composition, functional potential, and metabolic activity within the same ecological framework [18, 19]. In cliffside habitats such as those occupied by *T. sutchuenensis*, these small-scale gradients are expected to be particularly pronounced and ecologically relevant.

Here, we integrate metagenomics, metatranscriptomics, transcriptomics, and metabolomics to investigate the organization of root-associated bacterial communities and their functional attributes across vertical soil layers and root-associated compartments in *T. sutchuenensis*. We specifically address the following questions: How do bacterial diversity and community composition vary across vertical gradients and root-associated niches? How do bacterial functional potential, gene expression, and metabolite profiles related to carbon, nitrogen, and phosphorus cycling vary across these spatial gradients? By linking community structure with functional and metabolic profiles, this study aims to provide a more integrated understanding of how belowground bacterial systems are organized across spatial scales. These insights will contribute to a clearer understanding of plant–microbe interactions in environmentally constrained habitats and support conservation and restoration strategies for this endangered species.

## Materials and methods

### Plant survey and soil sampling

A comprehensive field investigation was carried out across all documented *T. sutchuenensis* populations within the geographic range of 108.7623°E to 108.8100°E and 31.7135°N to 31.7095°N, based on records from the National Specimen Information Infrastructure (http://www.nsii.org.cn) and recent local botanical surveys (Table S1). A total of nine individual trees were selected as biological replicates for metagenomic, metatranscriptomic, and transcriptomic analyses, and all samples were consistently collected from these same individuals to ensure comparability across compartments and omics datasets. For metabolomic analysis, an expanded set of 18 individual trees was included to increase statistical robustness and ensure reliable detection of metabolic variation. All sampled trees were healthy adults of similar age and height. All sampling was conducted during the active growing season (25–26 May 2024), when root systems are metabolically active, to minimize potential bias associated with seasonal dormancy and root turnover. For each tree, root systems were exposed using careful manual excavation with trenching around the base of the tree, revealing three vertical depth layers (0–20, 20–40, and 40–60 cm). Each depth layer was sampled independently from the same individual tree, ensuring matched vertical profiles within plants. Only fine roots (diameter < 2 mm), which are primarily responsible for nutrient uptake and microbial interactions, were selected for downstream analyses, while coarse structural roots were excluded. Roots were carefully excavated using a narrow trench spade and hand tools to minimize disturbance.

Rhizosphere soil was defined as the soil tightly adhering to root surfaces; loosely attached soil was first gently shaken off and discarded, and the remaining adhering soil was carefully collected using sterilized spatulas. Bulk soil was collected at the same depth intervals as the corresponding root samples (i.e. depth-matched), at least 10 cm horizontally away from the root zone, to avoid rhizosphere influence while maintaining vertical comparability. All soil samples were placed in sterile 50 ml centrifuge tubes, immediately transported on ice to the laboratory, and stored at −80°C for later analysis. To characterize microbial communities associated with the root surface, the episphere was obtained by rinsing roots in sterile water and gently scraping the outer epidermal layer with a sterile scalpel. To isolate the endosphere, root segments were surface-sterilized by sequential washing in 70% ethanol (1 min), 2.5% sodium hypochlorite (3 min), and three sterile-water rinses, after which the sterilized tissues were flash-frozen in liquid nitrogen and ground to a fine powder (Fig. S1).

### Root and soil physicochemical properties

Root samples were thoroughly rinsed with distilled water to remove adhering soil particles and debris without damaging fine structures. High-resolution images of the cleaned roots were captured using a flatbed scanner under consistent lighting and magnification. These images were analyzed using ImageJ software, with calibrated pixel-to-length conversion, to determine morphological traits including total root length, projected area, surface area, and average diameter. Specific root length (SRL) was calculated as the ratio of root length to dry mass (mg^−1^). Fresh root biomass was measured immediately after washing using a precision electronic balance (±0.001 g). To assess dry biomass, roots were placed in paper envelopes, oven-dried at 70°C for 72 h to constant weight, and then reweighed. For elemental analysis, dried root samples were finely ground using a ball mill. Total carbon (C) and nitrogen (N) content were quantified using a CHN elemental analyzer (Vario EL III). Total phosphorus (P) and potassium (K) were measured after microwave-assisted acid digestion (typically using a mixture of HNO_3_ and HClO_4_) and analyzed using inductively coupled plasma optical emission spectrometry (ICP-OES, PerkinElmer Optima 8000), following the protocol described previously [20, 21].

Soil pH was determined using a soil–water suspension (1:2.5 ratio) by mixing 10 g of air-dried, 1 mm-sieved soil with 25 ml of deionized water. After 30 min of standing, the supernatant was measured using a calibrated glass electrode pH meter (model PHS-2F). Soil organic matter (SOM) was assessed via the classical dichromate oxidation method. Specifically, 0.1–0.5 g of finely ground soil (<0.25 mm) was digested in 10 ml of 0.36 mol/l K_2_Cr_2_O_7_–H_2_SO_4_ solution, heated to 185°C–190°C to ensure complete oxidation, and then titrated with standardized FeSO_4_ to quantify the amount of oxidized organic carbon. Soil moisture content was calculated gravimetrically by weighing fresh field-collected soil, drying at 105°C for 12 h, cooling in a desiccator, and recording the final weight to determine water loss percentage [20, 21].

### Metagenomic sequencing analysis

To investigate the taxonomic and functional composition of the soil microbiome associated with *T. sutchuenensis*, 108 samples were collected from four distinct bacterial compartments: bulk soil, rhizosphere, episphere, and endosphere (9 replicates × 3 depth layers × 4 compartments) (Fig. S1). Although shotgun sequencing captures multiple microbial domains, downstream analyses in this study focused on bacteria due to their dominant representation and higher annotation reliability across samples, enabling more robust taxonomic and functional comparisons. Total genomic DNA was extracted using the Qiagen DNeasy PowerSoil Kit (Qiagen, USA), and the quality was assessed using a NanoDrop spectrophotometer (Thermofisher, CA, USA) and agarose gel electrophoresis. High-quality DNA (≥1 μg per sample) was used to prepare sequencing libraries using the NEBNext® Ultra™ II DNA Library Prep Kit for Illumina (New England Biolabs, USA), following the manufacturer’s protocol. DNA fragmentation was performed via Covaris ultrasonicator (Covaris, MA, USA) to an average size of ~350 bp. Subsequent steps included end repair, A-tailing, adapter ligation, and PCR enrichment. Libraries were purified using AMPure XP beads (Beckman Coulter, USA) and assessed for quality and size distribution using an Agilent 2100 Bioanalyzer (Agilent, CA, USA) and Qubit fluorometer (Thermofisher, CA, USA). Sequencing was carried out on an Illumina NovaSeq 6000 platform in paired-end 150 bp (PE150) mode. Raw reads were trimmed and quality-controlled using fastp (v0.23.2) to remove low-quality sequences and adapters. To minimize host contamination, reads originating from plant DNA were identified and removed prior to downstream analyses, and only non-host reads were retained for microbial community profiling. Taxonomic annotation of bacterial communities was performed using the Kraken2 classifier with the GTDB (Genome Taxonomy Database) as reference. Functional profiling was conducted by mapping gene predictions to eggNOG and KEGG Orthology databases using eggNOG-mapper (v5.0). To identify microorganisms associated with limonene degradation, we annotated metagenomic contigs with KEGG Orthologs related to the limonene degradation pathway (ko00903) and assigned taxonomy to the corresponding genes using Kaiju and Kraken2. Diversity metrics, including alpha diversity (Chao1 richness and Shannon index) and beta diversity Principal Coordinates Analysis (PCoA) based on Bray–Curtis dissimilarity), were calculated to assess community complexity and composition. Differences in community composition among compartments and depths were further evaluated using permutational multivariate analysis of variance (PERMANOVA) implemented in the “adonis” function of the vegan package in R (999 permutations). To explore co-occurrence patterns among genera, Spearman correlation networks were constructed (*r* > 0.75, adjusted *P* < .01, Benjamini–Hochberg correction). Network topologies were visualized using the “igraph” and “Hmisc” packages in R and rendered in Gephi (v0.10.1) for community module detection. To determine the ecological roles of nodes, within-module connectivity (Zi) and among-module connectivity (Pi) were calculated, and nodes were classified into four categories: network hubs (Zi > 2.5, Pi >0.62), module hubs (Zi > 2.5, Pi ≤0.62), connectors (Zi ≤ 2.5, Pi >0.62), and peripherals (Zi ≤ 2.5, Pi ≤0.62). Differentially abundant taxa across compartments and depths were identified using Linear Discriminant Analysis Effect Size (LEfSe), with Linear Discriminant Analysis score (LDA > 3) and *P* < .05 using the “lefser” package.

### Plant transcriptomic sequencing analysis

For transcriptome profiling of *T. sutchuenensis* root tissues, a total of 27 root samples (three groups, each with nine replicates) were collected, representing three vertical depth layers (0–20, 20–40, and 40–60 cm), with nine samples per layer. Clean root fragments were snap-frozen in liquid nitrogen in the field within 3 min of collection using a liquid nitrogen dry shipper and then stored at −80°C until RNA extraction. All tools were flame-sterilized between samples to avoid cross-contamination. For RNA extraction, 100 mg of frozen root tissue per sample was used. Samples were homogenized under liquid nitrogen using a prechilled mortar and pestle until a fine powder was obtained to ensure complete cell disruption and preservation of RNA integrity. Total RNA was extracted using the Qiagen RNeasy Plant Mini Kit, following the manufacturer’s protocol, and RNA quality was assessed using an Agilent 2100 Bioanalyzer [22]. High-quality RNA samples were subjected to Illumina sequencing for transcriptomic analysis. Raw sequencing data were subjected to stringent quality control, including the removal of low-quality reads and adapter sequences. Error rates, Q20 and Q30 scores (percentage of bases with a quality score above 20 and 30, respectively), and GC content were assessed to ensure sequencing accuracy. A total of 589 102 high-quality clean reads were assembled *de novo* using the Trinity software package, producing a comprehensive reference transcriptome. Functional annotation of assembled unigenes was performed by mapping against multiple public databases, including Gene Ontology (GO), Kyoto Encyclopedia of Genes and Genomes (KEGG), Clusters of Orthologous Groups (COG), the National Center for Biotechnology Information (NCBI) nonredundant nucleotide (Nt) and protein (Nr) databases, Swiss-Prot, and Pfam protein family databases. To explore regulatory mechanisms, transcription factors (TFs) were predicted based on homology searches against the PlantTFDB database. Differentially expressed genes (DEGs) were identified using the DESeq2 package in R (https://www.r-project.org/), applying thresholds of |log₂(fold change)| > 2 and an adjusted *P*-value <.05 to define significant expression changes. Functional enrichment analyses of DEGs were conducted based on GO terms and KEGG pathway classifications using the “clusterProfiler” and “enrichplot” packages in R.

### Soil metatranscriptomic sequencing analysis

To investigate the active bacterial functions in contrasting soil microenvironments, a total of 54 soil samples were collected for metatranscriptomic analysis, comprising 27 rhizosphere samples and 27 bulk soil samples, evenly distributed across three vertical depth layers (0–20, 20–40, and 40–60 cm) with nine biological replicates per layer. All samples were snap-frozen in a liquid nitrogen dry shipper and transported to the lab, where they were stored at −80°C until RNA extraction. Total RNA was extracted using the Qiagen RNeasy PowerSoil Total RNA Kit, followed by DNase I treatment to eliminate residual genomic DNA. RNA quality and integrity were assessed using an Agilent 2100 Bioanalyzer, and only samples with RIN ≥ 7.0 were retained. Paired-end sequencing (2 × 150 bp) was carried out on the Illumina HiSeq™ 4000 platform, generating raw reads ~300 bp in length from both ends. To ensure accuracy and data reliability, raw sequences were processed using Sickle (v1.33) and SeqPrep, which filtered out low-quality reads, short fragments, and adapter-contaminated sequences based on default quality thresholds. Gene prediction from clean sequences was performed using MetaGeneMark, which identifies protein-coding regions from metagenomic and metatranscriptomic data. The predicted open reading frames were translated into amino acid sequences and subsequently annotated through alignment against the KEGG database to assign functional categories and infer metabolic pathways.

### Metabolomic sequencing analysis

A total of 162 samples, including 54 for rhizosphere, 54 for bulk soil, and 54 for roots (three vertical layers, each with 18 replicates), were collected for untargeted metabolomics. Each sample was placed into pre-cooled sterile 2 ml microcentrifuge tubes and immediately flash-frozen in liquid nitrogen on-site. In the lab, samples were ground to a fine powder under liquid nitrogen using prechilled mortars and pestles. For metabolite extraction, 100 mg of homogenized material was mixed with 1 ml of ice-cold 80% methanol (Liquid Chromatography–Mass Spectrometry (LC-MS) grade) containing 0.1% formic acid, vortexed for 1 min, and sonicated on ice for 10 min. The mixture was then centrifuged at 13 000 *xg* for 15 min at 4°C, and the supernatant was transferred to clean tubes and stored at −80°C prior to analysis. Before injection into the Liquid Chromatography–Tandem Mass Spectrometry (LC-MS/MS) system, 400 μL of extract was thawed and diluted with LC-MS-grade water to reach a final methanol concentration of 53%. The diluted samples were again centrifuged at 15 000 *xg* for 20 min at 4°C to remove particulates. Metabolite separation and detection were performed using a Vanquish UHPLC system (Thermo Fisher Scientific, Germany) coupled to a high-resolution Orbitrap Q Exactive™ HF mass spectrometer (Thermo Fisher Scientific, Germany). Hydrophilic interaction liquid chromatography (HILIC) was used to retain and separate polar metabolites, and electrospray ionization (ESI) was applied in both positive and negative modes. Metabolite ions were acquired using data-dependent MS/MS scanning in a triple quadrupole (QqQ) configuration. Raw spectral data were processed using Compound Discoverer 3.1 (CD3.1, Thermo Fisher Scientific), which performed automated peak detection, alignment, deconvolution, and quantification. Identified metabolites were annotated by matching features against KEGG, Human Metabolome Database (HMDB), and LIPIDMaps. Multivariate statistical techniques, including Principal Component Analysis (PCA) and Orthogonal Partial Least Squares Discriminant Analysis (OPLS-DA), were implemented using the MetaX software package (v1.0.4) to visualize metabolic variation across sample groups and detect clustering patterns. Univariate statistical tests (two-tailed Student’s *t-*test) were applied to identify differentially expressed metabolites (DEMs), with filtering thresholds set at VIP > 1, *P*-value <.05, and fold change ≥2 or ≤ 0.5. To identify key biomarkers and drivers of group separation, a random forest classification model was built using the “randomForest” package in R.

### Statistical analyses

All statistical analyses were performed using GraphPad Prism 8 (GraphPad Software, Inc., USA). One-way analysis of variance (ANOVA) was employed to compare means across multiple groups, while independent two-sample *t*-tests were used for pairwise comparisons. When ANOVA indicated significant differences, *post hoc* multiple comparisons were performed using Tukey’s honestly significant difference test to identify differences between specific groups. Results were reported as mean ± standard error (SE), and statistical significance was denoted using the following thresholds: *P* < .05 (^*^), *P* < .01 (^**^), and *P* < .001 (^***^). All figures were further refined and formatted for publication using Adobe Illustrator CC, ensuring consistency in layout, color schemes, and label formatting across panels.

## Results

### Soil physicochemical properties differ significantly between bulk and rhizosphere soils

**Figure 1:**
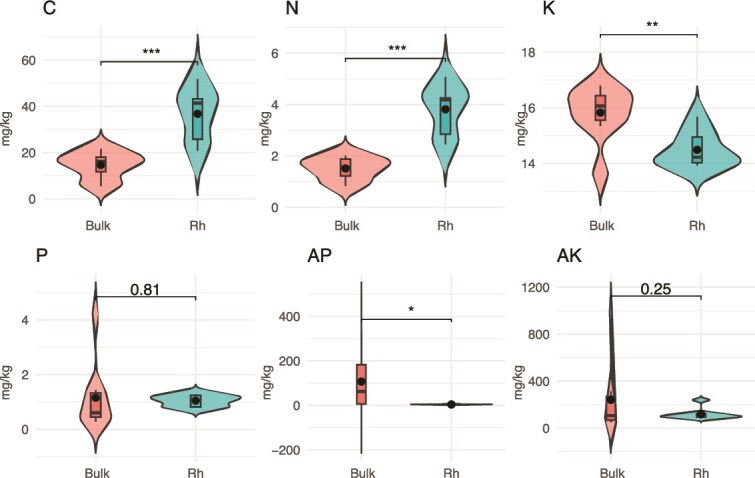
Comparisons of soil physicochemical properties between bulk soil and rhizosphere soils in *T. sutchuenensis.* Boxplots display concentrations of total carbon (C), total nitrogen (N), total potassium (K), total phosphorus (P), available phosphorus (AP), and available potassium (AK) in bulk soil (Nr) and rhizosphere (Rh) compartments. *P*-values from statistical tests are indicated above each plot. Asterisks denote significance levels: *P* < .05 (^*^), *P* < .01 (^**^), *P* < .001 (^***^).

Analysis of soil nutrient profiles revealed distinct differences between bulk and rhizosphere soils (Fig. 1). Soil C and N contents were both significantly higher in the rhizosphere compared with the bulk soil (*P* = .00018 for C, *P* = 3.3e–05 for N). Soil K (*P* = .0036) and AP (*P* = .038) levels were elevated in bulk soils. In contrast, total P did not show a statistically significant difference between bulk and rhizosphere soils (*P* = .81). Similarly, AK exhibited lower concentrations in rhizosphere soils than in bulk soils (*P* = .25), though the difference was not statistically significant at the 0.05 threshold.

### Root morphological and physiological traits vary significantly with soil depth

**Figure 2:**
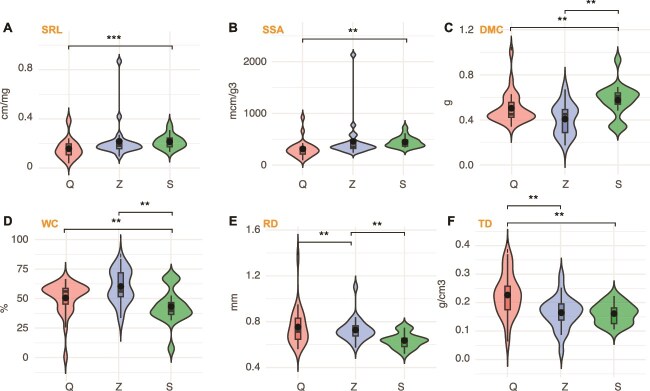
Root morphological and physiological trait variation across soil depth in *T. sutchuenensis*. Boxplots show depth-dependent changes in specific root length, specific surface area, dry matter content, root water content, average root diameter, and tissue density across three soil layers: 0–20 cm (Q), 20–40 cm (Z), and 40–60 cm (S). Asterisks denote significance levels: *P* < .05 (^*^), *P* < .01 (^**^), *P* < .001 (^***^).

Root traits of *T. sutchuenensis* varied significantly across soil depths, with SRL and specific surface area highest in the deep layer (S, 40–60 cm), followed by the middle (Z) and shallow (Q) layers (*P* < .01) (Fig. 2). Root dry matter content also showed a depth-related increase, with significantly greater values in the S layer compared to Q and Z (*P* < .05). Root water content peaked in the middle layer (Z, 20–40 cm), with both Q and S exhibiting lower values (*P* < .05). In contrast, root diameter and tissue density were significantly higher in the shallow layer (Q, 0–20 cm) than in the deeper layers (*P* < .05 and *P* < .001, respectively), highlighting distinct morphological adjustments along the vertical profile.

### Distinct bacterial diversity, composition, and function across root-associated compartments

**Figure 3:**
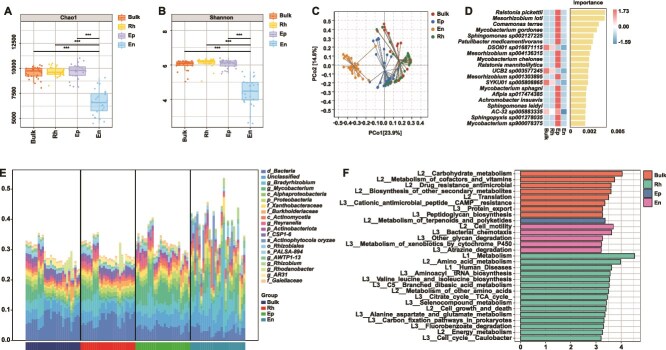
Metagenomic analysis of bacterial diversity, composition, and function across bulk, rhizosphere (Rh), root episphere (Ep), and endosphere (En) in *T. sutchuenensis*. (A, B) Alpha diversity indices (Chao1 and Shannon) show a significant decline in richness and diversity from bulk soil to root compartments. (C) Principal coordinate analysis (PCoA) based on Bray–Curtis dissimilarity reveals distinct clustering by compartment. (D) Random forest analysis identifies key bacterial taxa contributing to differentiation among compartments. Heatmap is shown as Z-score–normalized values, where red indicates higher relative abundance and blue indicates lower relative abundance. (E) Linear discriminant analysis (LDA) identifies biomarker taxa significantly enriched in each group (LDA score > 3). (F) Taxonomic contribution to limonene degradation pathway (ko00903).

Rarefaction curves confirmed adequate sequencing depth across all compartments, with sufficient coverage to capture the majority of bacterial taxa present (Fig. S2A). A total of 30 041 bacterial taxa were shared among all four compartments (Fig. S2B), while each compartment also harbored unique taxa, reflecting distinct bacterial assemblages. Alpha diversity metrics did not show a gradual decreasing trend across compartments. Instead, a significant reduction was observed specifically in the endosphere. Both the Chao1 index (Fig. 3A) and the Shannon index (Fig. 3B) were significantly higher in bulk soil, the rhizosphere, and the episphere compared to the endosphere (*P* < 0.001). PCoA based on Bray–Curtis dissimilarity showed separation among compartments (Fig. 3C), and this pattern was supported by PERMANOVA analysis, which confirmed significant differences in bacterial community composition among compartments (*P* < .01). However, partial overlap was observed between bulk soil and rhizosphere samples, reflecting their compositional similarity due to spatial proximity. The first two axes explained 23.9% and 14.8% of the total variance, respectively, indicating that additional factors contribute to the overall community structure. Random forest analysis identified key bacterial taxa that contributed most to distinguishing the four compartments (Fig. 3D). Notable contributors included *Ralstonia pickettii*, *Mesorhizobium loti*, and *Mycobacterium gordonae*, which were enriched in root-associated niches, particularly in the episphere. Taxonomic composition varied significantly at both the phylum and genus levels (Figs S2 and S3). Proteobacteria and Actinobacteriota dominated across all compartments, but their relative abundances shifted notably: Proteobacteria were enriched in the endosphere, while Actinobacteriota and Acidobacteriota were more prevalent in the episphere. At the genus level, *Bradyrhizobium*, *Mycobacterium*, and *Ralstonia* showed differential enrichment along the soil-to-root continuum. Analysis of carbohydrate-active enzymes (CAZymes) showed elevated levels of GT1 in endosphere, and glycoside hydrolases (e.g. GH23, GH3) in epispheric, bulk soil, and rhizospheric regions (Fig. S3C). Further KEGG pathway analysis highlighted enrichment of functions related to limonene degradation (ko00903, “Monoterpenoid degradation”), carbon fixation, and amino acid metabolism in episphere samples (Fig. S3D). Specific pathways such as bacterial chemotaxis, protein export, and antimicrobial resistance were also enriched in root-associated microbiota. Functional contributions to the limonene degradation pathway (ko00903) were dissected at the taxonomic level (Fig. 3E & Fig. S4), revealing that specific genera, including *Mycobacterium* and *Sphingomonas*, played dominant roles in this plant secondary metabolite transformation within the endospheric and epispheric microbiome. LDA revealed that each root-associated compartment exhibited functionally distinct bacterial communities, with specific metabolic pathways serving as key discriminative features. Notably, carbohydrate metabolism and carbon fixation pathways were enriched in bulk soil and rhizosphere samples, while the metabolism of terpenoids, polyketides, and xenobiotics was significantly associated with the episphere and endosphere microbiota (Fig. 3F).

We found that root-associated bacterial diversity and composition vary significantly across three vertical soil layers and root compartments, with the root endosphere consistently showing lower alpha diversity than other compartments (Fig. S5). Beta diversity analyses revealed clear stratification influenced by both depth and proximity to roots, highlighting spatial structuring of bacterial communities. Several taxa, including *R. pickettii*, *M. loti*, and *M. gordonae*, were identified as depth- and compartment-specific biomarkers driving this differentiation.

### Metatranscriptomic profiles reveal functional and taxonomic differences across soil compartments

Metatranscriptomic analysis revealed clear distinctions in bacterial gene expression patterns between bulk soil and rhizosphere soils. At the phylum level (Fig. S6A), bacterial groups such as Firmicutes and Gemmatimonadetes were significantly enriched in the rhizosphere compared to the bulk soil. At the genus level (Fig. S6B), taxa such as *Mesorhizobium*, *Bradyrhizobium*, and *Symbiodinium* exhibited significantly higher expression in rhizosphere samples. Functional annotation based on the KEGG database (Fig. S6C) showed enrichment of pathways involved in carbon fixation in photosynthetic organisms (ko00710), pantothenate and CoA biosynthesis (ko00770), and extracellular matrix (ECM)–receptor interaction (ko04112) in rhizosphere communities. Additionally, carbohydrate-active enzyme profiles (Fig. S6D) revealed significantly higher expression of glycoside hydrolase (GH) and glycosyltransferase (GT) families, such as GH152, GT61, and GH1, in the rhizosphere. For vertical layers, dominant phyla such as Proteobacteria and Actinobacteria exhibited reduced expression in upper soil layers, while genera like *Entotheonella*, *Granulicella*, and *Clavibacter* showed depth-specific transcriptional patterns (Fig. S7). Functional gene expression also varied vertically, with GT13 glycoside hydrolases enriched in mid-depth soils and chaperonin GroES (K04078) more abundant at the surface.

### Root transcriptome analysis reveals depth-specific gene expression and functional differentiation

**Figure 4:**
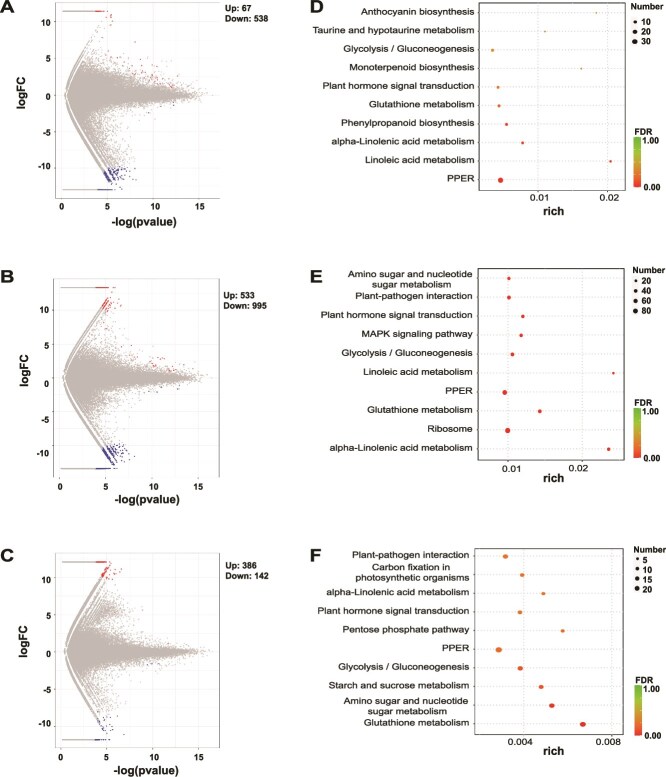
Metatranscriptomic and transcriptomic profiles across soil compartments and root depths in *T. sutchuenensis*. (A–C) Transcriptomic comparison of root tissues across three depth levels, Q (0–20 cm), Z (20–40 cm), and S (40–60 cm). (D–F) KEGG pathway enrichment analysis of DEGs from Q vs. Z (D), Q vs. S (E), and Z vs. S (F) comparisons.

As shown in Fig. S8, most unigenes exceeded 200 bp, with many surpassing 500 bp, indicating a high-quality transcriptome assembly suitable for functional annotation, and transcription factor analysis revealed 63 families, predominantly bHLH, MYB-related, NAC, ERF, and C2H2 (Fig. S9). A total of 605 DEGs were identified between Q vs. Z, including 67 upregulated and 538 downregulated genes (Fig. 4A). In the Q vs. S comparison, 1528 DEGs were detected (533 upregulated and 995 downregulated; Fig. 4B), while Z vs. S revealed 528 DEGs (386 upregulated and 142 downregulated; Fig. 4C). KEGG enrichment analysis of DEGs showed consistent enrichment in metabolic and stress-related pathways. In the Q vs. Z group (Fig. 4D), DEGs were significantly associated with linoleic acid metabolism, plant hormone signaling, and phenylpropanoid biosynthesis. The Q vs. S comparison showed enrichment in ribosomal activity, mitogen-activated protein kinase (MAPK) signaling, and plant–pathogen interactions (Fig. 4E). In the Z vs. S comparison, enriched pathways included starch and sucrose metabolism, carbon fixation, and glutathione metabolism (Fig. 4F).

### Distinct metabolomic profiles between rhizosphere and bulk soils and among root depths

**Figure 5:**
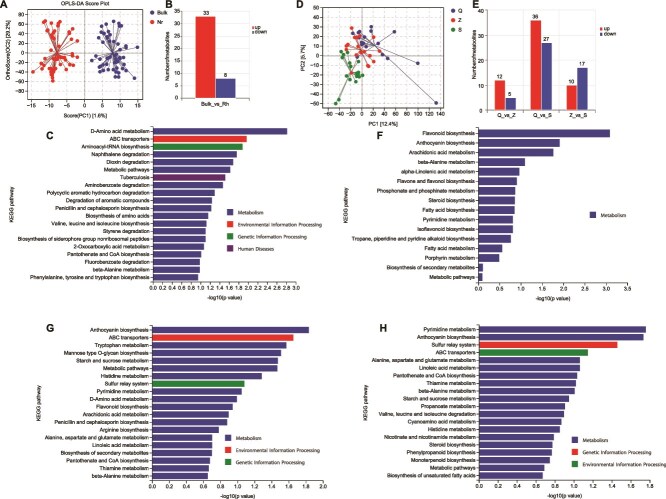
Untargeted metabolomic analysis of soil and root microenvironments in *T. sutchuenensis*. (A) OPLS-DA score plot showing clear separation between bulk soil and rhizosphere (Rh) soil metabolomes. (B) Bar plot of differentially expressed metabolites (DEMs) between bulk and rhizosphere soils. (C) PCA plot of root metabolomes across three depth layers—Q (0–20 cm), Z (20–40 cm), and S (40–60 cm). (D) Numbers of upregulated and downregulated DEMs in the pairwise comparisons Q vs. Z, Q vs. S, and Z vs. S. (E) KEGG pathway enrichment of DEMs between bulk and rhizosphere soil. (F–H) KEGG functional enrichment of root DEMs in Q vs. Z (F), Q vs. S (G), and Z vs. S (H) comparisons.

OPLS-DA demonstrated clear separation between the two groups, indicating distinct metabolic signatures driven by root influence (Fig. 5A). In total, 41 DEMs were identified, including 33 upregulated and 8 downregulated compounds in the rhizosphere compared to bulk soil (Fig. 5B). KEGG pathway enrichment analysis of these DEMs revealed functional enrichment in several key metabolic processes (Fig. 5C). These included *D*-amino acid metabolism, ATP-binding cassette (ABC) transporters, xenobiotic degradation (e.g. dioxin and naphthalene degradation), and amino acid biosynthesis (e.g. valine, leucine, and isoleucine pathways).

### Root metabolomes exhibit depth-dependent divergence in composition and function

PCA of root metabolomic data showed distinct clustering of samples from Q (0–20 cm), Z (20–40 cm), and S (40–60 cm) layers, with PC1 and PC2 accounting for 12.4% and 5.7% of the total variance, respectively (Fig. 5D). Comparative analysis revealed a total of 17 DEMs between Q vs. Z, 63 DEMs between Q vs. S, and 27 DEMs between Z vs. S, with both up- and downregulated metabolites identified in each pairwise comparison (Fig. 5E). KEGG-based functional enrichment of these depth-related DEMs revealed unique biological processes enriched at different depths. In the Q vs. Z comparison (Fig. 5F), significant pathways included flavonoid and anthocyanin biosynthesis, arachidonic acid metabolism, and fatty acid biosynthesis. In Q vs. S (Fig. 5G), enriched pathways included tryptophan metabolism, ABC transporters, and plant hormone biosynthesis. The Z vs. S comparison (Fig. 5H) showed enrichment in pyrimidine metabolism, sulfur relay systems, and amino acid pathways.

### Coordinated multi-omics insights into carbon, nitrogen, and phosphorus metabolism

**Figure 6:**
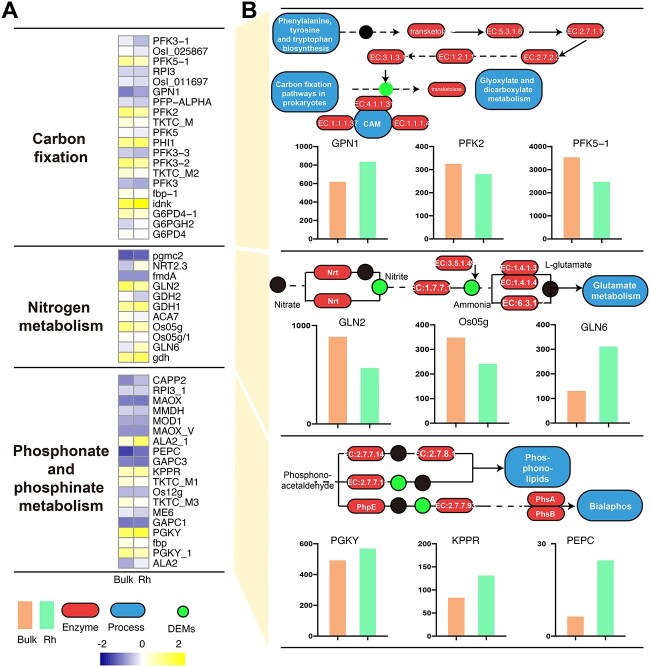
Heatmaps and pathway illustrations highlighting key genes and metabolites involved in carbon fixation, nitrogen metabolism, and phosphonate and phosphinate metabolism in bulk and rhizosphere. (A) Heatmaps of selected KEGG orthologs related to each pathway across bulk and rhizosphere, shown as Z-score-normalized values (yellow: higher abundance; blue: lower abundance). (B) Schematic pathway maps showing enzymatic steps and metabolic connections; red shapes denote altered enzymes, green circles denote metabolites, and blue boxes denote associated metabolic pathways.

Integrated multi-omics analyses revealed coordinated shifts in carbon, nitrogen, and phosphorus metabolism between rhizosphere and bulk soils (Fig. 6). In the carbon cycle, transcriptomic changes in genes such as *PFK2*, *GAPC3*, *PEPC*, and *GPN1* indicated significant modulation of carbon fixation pathways. For nitrogen metabolism, transcripts including GLN2, *GLN6*, and *GDH1/2* were differentially expressed and corresponded with altered concentrations of nitrite- and ammonia-associated metabolites. In phosphorus cycling, key genes such as *PGKY*, *KPPR*, and *PhpE*, involved in phosphonolipid biosynthesis and bialaphos metabolism, were significantly affected, highlighting rhizosphere-specific regulation of phosphorus turnover.

### Co-occurrence analysis reveals modular structure and key bacterial hubs

**Figure 7:**
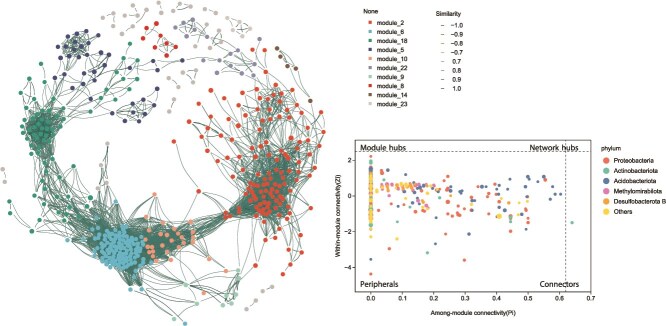
Co-occurrence network analysis of bacterial taxa across spatial scales in *T. sutchuenensis* root-associated habitats. The network was constructed using significant Spearman correlations (*r* > 0.75, adjusted *P* < .01) among bacterial genera and reveals 498 nodes (taxa) and 66 886 edges (co-occurrence relationships). Z–P plot (within-module connectivity Zi vs. among-module connectivity Pi) classifies bacterial nodes into topological roles: peripherals, connectors, module hubs, and network hubs.

To explore the ecological interactions among bacterial taxa at a spatial resolution, a co-occurrence network was constructed based on significant Spearman correlations among bacterial genera. The resulting network exhibited a high degree of complexity, comprising 498 nodes (bacterial taxa) and 66 886 edges, with a modularity score of 0.1385, indicating a moderately modular structure with several distinct clusters (modules) (Fig. 7 & Table S2). Topological analysis of the network revealed high clustering (transitivity = 0.8735) and moderate density (0.5405), suggesting strong interconnectivity among taxa. The average path length was 1.65, and the network had a diameter of 5.47, reflecting efficient information transfer among nodes. Notably, Proteobacteria, Actinobacteriota, and Acidobacteriota were the most dominant phyla across all modules, highlighting their central roles in root-associated bacterial networks. Most nodes were categorized as peripherals (low connectivity), whereas a smaller subset functioned as module hubs and connectors. Functionally, distinct modules likely represent coordinated groups of microbes responding to local environmental cues or performing complementary ecological roles (e.g. nutrient cycling, stress tolerance, or metabolite degradation).

### Bacterial diversity correlates with soil nutrient availability at spatial scales

Correlation analysis revealed strong negative associations between bacterial alpha-diversity (Chao1 and Shannon indices) and multiple soil chemical properties, including AP, P, AK, and K, with the strongest negative correlation observed between Chao1 and N (*r* = −0.73) (Fig. S10). In contrast, positive correlations were observed among most soil nutrient variables, particularly between AK and P (*r* = 0.94), AK and AP (*r* = 0.85), and P and AP (*r* = 0.78). The first MDS axis (MDS1) was positively correlated with soil potassium and K (*r* = 0.72) but negatively with bacterial diversity.

## Discussion

Our results demonstrate that root morphological traits and soil physicochemical properties display pronounced vertical stratification, underscoring the highly localized nature of plant–soil interactions in *T. sutchuenensis* habitats. Interestingly, the observed pattern of increasing SRL and decreasing root diameter with depth contrasts with the conventional expectation that deeper roots are thicker and primarily serve structural functions. Similar patterns have been reported in other woody species [23], suggesting that root trait variation along depth gradients may be strongly context-dependent. From the perspective of the root economics spectrum (RES), higher SRL is generally associated with acquisitive strategies characterized by faster resource uptake but lower tissue investment, whereas higher root dry matter content reflects more conservative strategies with greater structural investment and longer lifespan [24, 25]. In this study, the coexistence of increased SRL and higher root dry matter content at depth suggests a decoupling of classical RES trait relationships, potentially reflecting adaptive responses to the unique environmental constraints of cliffside habitats. We therefore interpret this pattern as indicating that deeper roots in *T. sutchuenensis* may balance resource acquisition efficiency and structural persistence, rather than strictly conforming to a single acquisitive or conservative strategy. The vertical shift in root traits corresponded closely with changes in rhizosphere soil properties. Specifically, we observed a significant enrichment of carbon, nitrogen, and available phosphorus in the rhizosphere relative to bulk soil. This enrichment likely arises from the combined effects of root-derived inputs, microbial turnover, and physicochemical processes occurring at the root–soil interface, rather than a single mechanism. While these patterns are consistent with enhanced microbial processing in the rhizosphere, our study focuses on static measurements and does not directly quantify dynamic processes such as root exudation, bacterial activity rates, or nutrient fluxes. This localized nutrient enrichment provides a crucial resource pool for root uptake and may play a central role in sustaining plant growth under the challenging cliffside conditions where *T. sutchuenensis* occurs.

The soil-to-root continuum showed compartment-specific differences in bacterial community structure, diversity, and function, rather than a continuous gradient. Alpha diversity metrics (Chao1 and Shannon indexes) were significantly lower in the endosphere compared to non-rhizosphere soil, rhizosphere, and episphere. This phenomenon is consistent with the two-step selection model described previously, where soil acts as a microbial reservoir, but only specific taxa successfully colonize the rhizosphere, episphere, and root interior [26, 27]. Notably, Proteobacteria and Actinobacteriota were dominant across compartments, but genera such as *Bradyrhizobium*, *Rhodanobacter*, and *Ralstonia* showed compartment-specific enrichment, pointing to niche adaptation and host selection pressure. These taxa are widely reported to be functionally associated with plant–soil interactions: *Bradyrhizobium* is known for its role in nitrogen cycling and plant-associated symbioses [28], *Ralstonia* includes taxa adapted to root environments with capabilities for metabolizing diverse carbon substrates [29], and *Rhodanobacter* has been linked to nitrogen transformation and adaptation to variable soil conditions [30]. Their enrichment in root-associated compartments suggests active involvement in nutrient transformation and utilization of root-derived compounds in this system. Random forest analysis and LDA further identified key indicator taxa and functional groups associated with each niche, reinforcing the role of habitat filtering. Compared to previous work in temperate and tropical forests [31, 32], our study provides insight into bacterial community turnover at sub-meter vertical scales. Importantly, KEGG and CAZyme functional annotations suggest a shift toward bacterial traits favoring host interaction, xenobiotic degradation, and carbohydrate metabolism near roots, revealing how microbes adaptively respond to root-derived substrates and spatial gradients in resource availability.

Transcriptome profiling of *T. sutchuenensis* roots at different depths uncovered distinct gene expression signatures reflective of adaptive physiological responses. Surface roots upregulated genes involved in hormone signaling, flavonoid biosynthesis, and defense-related pathways such as MAPK signaling and plant–pathogen interaction, suggesting active responses to environmental stimuli and bacterial encounters [33]. In contrast, deeper roots showed enhanced expression of genes related to core metabolism, carbon fixation, and antioxidant responses, indicating a shift toward maintenance and resilience functions in resource-limited conditions [34]. These findings resonate with studies in other woody plants where root zone–specific gene expression mediates nutrient foraging, stress tolerance, and microbiome interaction [35, 36]. Moreover, the DEGs identified across Q vs. Z, Q vs. S, and Z vs. S comparisons displayed functional enrichment in linolenic and linoleic acid pathways, reinforcing the depth-related reprogramming of lipid signaling and membrane composition. These pathways play central roles in plant stress signaling and membrane dynamics, as linolenic acid is a precursor for jasmonic acid biosynthesis, a key regulator of defense and environmental responses, while linoleic acid contributes to maintaining membrane fluidity and stability under variable conditions [37, 38]. Their enrichment is consistent with depth-associated adjustments in lipid-mediated signaling and membrane properties in response to environmental variation, such as changes in moisture, oxygen availability, and microbial interactions [39]. Such patterns indicate that lipid-related processes may contribute to how *T. sutchuenensis* roots adjust to heterogeneous cliffside environments, where environmental variability requires coordinated regulation of stress signaling and cellular stability. Together, these results highlight transcriptomic differentiation along vertical gradients, which may underlie the root system’s ability to acclimate to the heterogeneous conditions of its cliffside habitat.

Our metabolomic analysis revealed that both soil and root microhabitats harbor chemically distinct environments that reflect underlying biological processes. Rhizosphere soils were metabolically enriched relative to bulk soil, with upregulation of metabolites involved in ABC transporters, amino acid biosynthesis, and xenobiotic degradation. These features are consistent with heightened bacterial activity and substrate turnover in the rhizosphere, a phenomenon reported across diverse systems [40]. Root metabolomes also varied significantly with depth. Surface roots displayed higher abundances of flavonoids, anthocyanins, and lipid derivatives—compounds often implicated in signaling, defense, and bacterial modulation [41]. Deeper roots, on the other hand, accumulated metabolites associated with primary metabolic processes such as amino acid and pyrimidine metabolism. KEGG pathway enrichment further highlighted the dynamic balance between primary and secondary metabolism in roots as a function of environmental depth, suggesting that *T. sutchuenensis* leverages distinct metabolic programs to optimize root–soil–microbe interactions across vertical gradients.

Integrative network and correlation analyses provided additional insight into the complexity of bacterial interactions and their relationships with environmental parameters. Co-occurrence network construction revealed a moderately modular structure with tightly clustered bacterial groups, dominated by Proteobacteria and Actinobacteriota. Within-module hubs and connectors were identified as potential keystone taxa, playing critical roles in maintaining community cohesion and cross-module communication. These findings are consistent with global soil microbiome networks, where modularity underpins resilience and functional redundancy [42, 43]. Furthermore, correlation analysis showed strong positive associations between soil carbon, nitrogen, and available phosphorus with bacterial diversity, while moisture and spatial gradients negatively impacted diversity. Such relationships reinforce the role of nutrient enrichment and spatial heterogeneity in shaping community assembly. This correspondence between soil conditions and bacterial richness echoes trends observed in alpine and desert soils [44–46] but offers a new resolution into how centimeter-scale gradients influence belowground biodiversity in forested cliffs.

Future work should focus on experimentally manipulating nutrient levels or bacterial consortia to verify causal relationships and expand to seasonal dynamics and functional validation (e.g. stable isotope tracing, metaproteomics). Given the ecological significance and conservation urgency of *T. sutchuenensis*, integrating processes into management strategies may enhance the success of restoration and *ex situ* conservation efforts. Our study not only advances fundamental knowledge of plant–soil–microbe interactions but also underscores the value of spatial precision in ecological genomics.

## Conclusion

In summary, our multi-omics investigation of *T. sutchuenensis* forests across spatial scales reveals a highly structured and interactive root–soil–microbe system. Root morphological, transcriptomic, and metabolomic traits are closely aligned with vertical gradients in soil nutrients and bacterial composition. Bacterial communities exhibit strong compartmentalization, functional specialization, and co-occurrence patterns that are tightly coupled to root influence and environmental microheterogeneity. These findings highlight the importance of high-resolution sampling in ecological studies and provide a valuable framework for understanding how rare and endangered species adapt to complex environments.

## Supplementary Material

Additional_figures_s1-s10_Table_S1-S2_ycag115

## Data Availability

The sequencing data were submitted to the Sequence Read Archive (SRA) database with BioProject accession number (MTBLS10710 and PRJNA1133092). Other data used to support the findings are available from the corresponding author upon request.
