# Supplementary material for: Spatial structuring of root-associated bacteria and metabolic landscapes in an endangered cliffside conifer Thuja sutchuenensis Franch
Source: ISME Commun. 2026 Apr 23;6(1):ycag115. doi: 10.1093/ismeco/ycag115 (PMC13196600; doi:10.1093/ismeco/ycag115)
Supplement: Additional_figures_s1-s10_Table_S1-S2_ycag115 [file additional_figures_s1-s10_table_s1-s2_ycag115.docx]

**Spatial structuring of root-associated bacteria and metabolic landscapes in an endangered cliffside conifer *Thuja sutchuenensis* Franch**

You-wei Zuo^1^*,* Yang Peng^1^, Wen-qiao Li^1^*,* Zhi-jang Yang^1^*,* Hong-ping Deng^1*^

^1^ Key Laboratory of Eco-Environment in the Three Gorges Reservoir Region, Ministry of Education, School of Life Sciences, Southwest University, 400715, Beibei, Chongqing, China

^*^ Corresponding author

Hong-ping Deng; E-mail: denghp@swu.edu.cn; Phone number: +86 13883395687


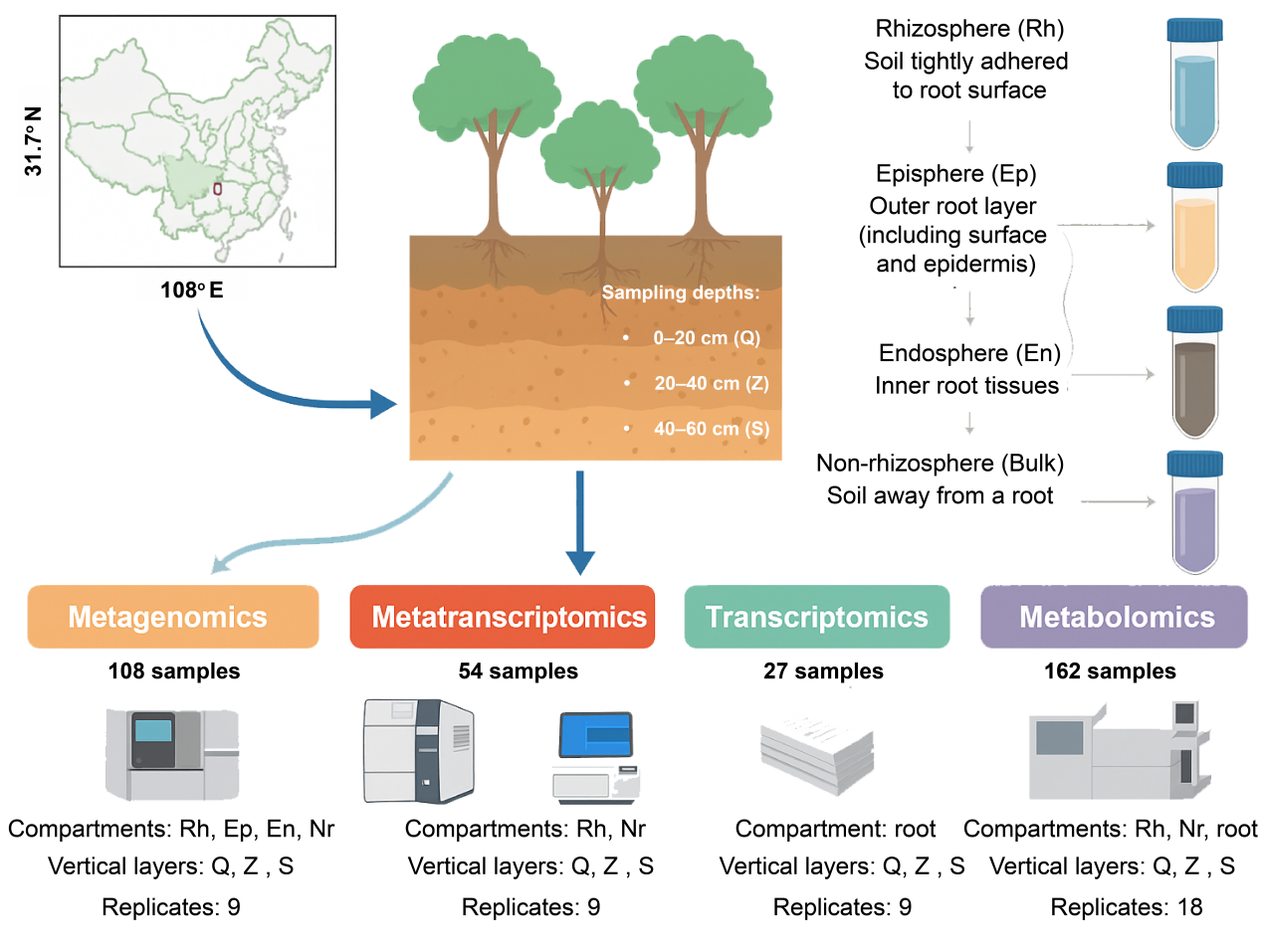


**Figure S1.Multi-omics experimental design to investigate soil–root interactions across spatial compartments and vertical layers in *Thuja sutchuenensis* populations.** Samples were collected across three vertical soil layers: 0–20 cm (Q), 20–40 cm (Z), and 40–60 cm (S). Four distinct microhabitats were sampled: non-rhizosphere (bulk soil), rhizosphere (Rh, soil tightly attached to roots), episphere (Ep, root surface), and endosphere (En, internal root tissues). These samples were subjected to integrated multi-omics analyses including metagenomics (108 samples), metatranscriptomics (54 samples), transcriptomics (27 samples), and metabolomics (162 samples).


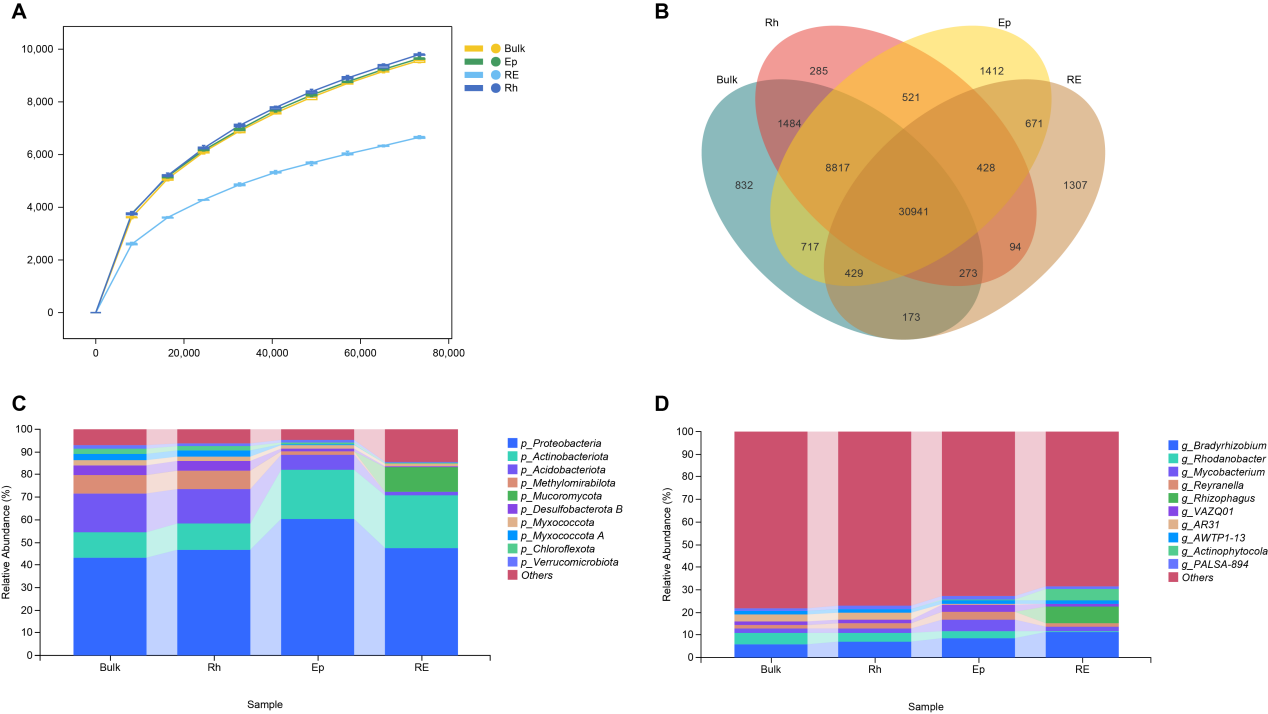


**Figure S2. Microbial diversity and community composition across four compartments: bulk, rhizosphere (Rh), root episphere (Ep), and root endosphere (RE).** (A) Rarefaction curves showing the sequencing depth and observed species richness in each group, indicating adequate sampling coverage. (B) Venn diagram illustrating the number of shared and unique microbial taxa across the four compartments, with a core microbiome of 30,041 taxa shared among all. (C & D) Taxonomic composition at the phylum level and genus-level.


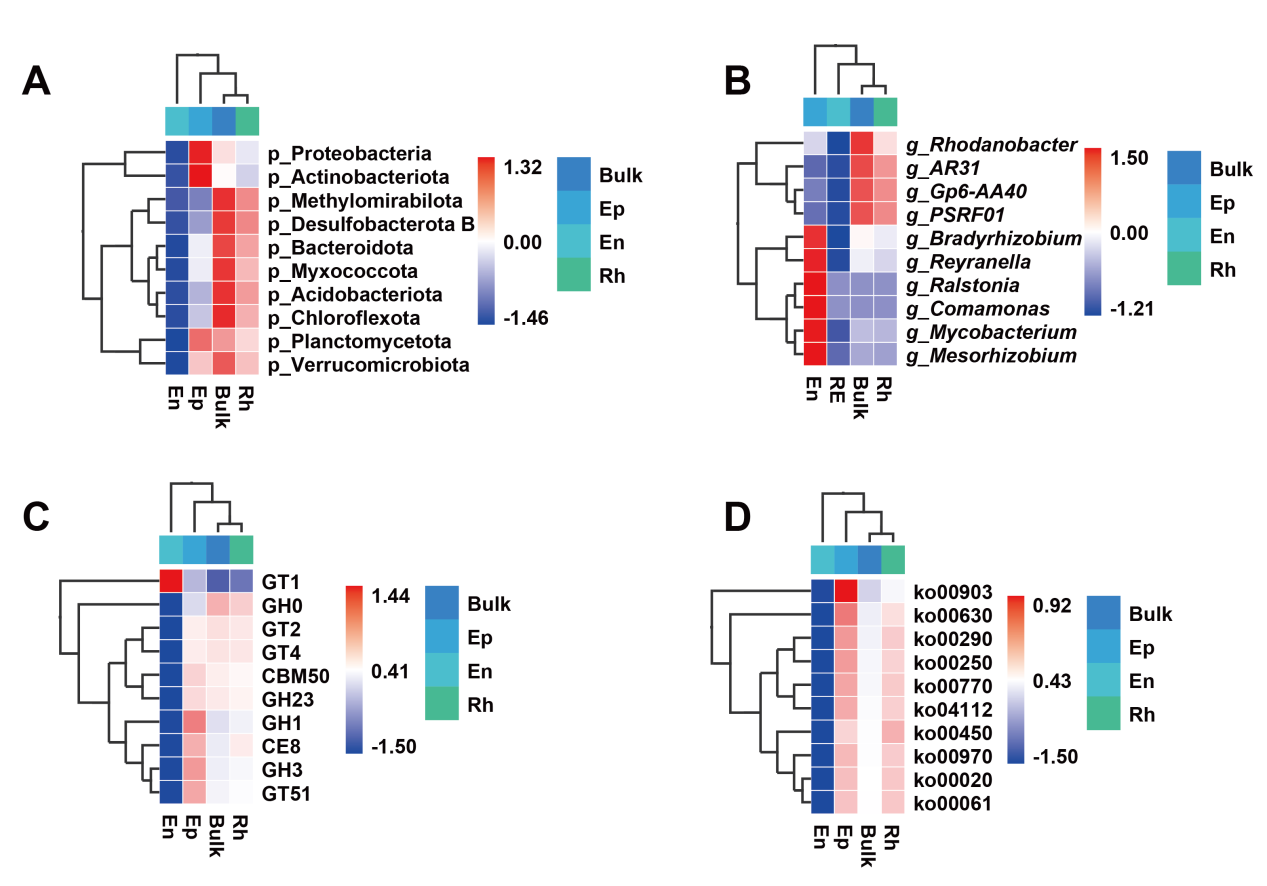


**Figure S3. Heatmap analysis of bacterial taxonomic composition and functional profiles across root-associated compartments.** (A) Relative abundance of dominant bacterial phyla; (B) relative abundance of selected bacterial genera; (C) distribution of carbohydrate-active enzyme (CAZyme) families; (D) KEGG ortholog functional profiles. Samples are grouped by compartment: bulk, rhizosphere (Rh), episphere (Ep), and endosphere (En). Heatmaps represent Z-score–normalized values, with red indicating higher relative abundance and blue indicating lower relative abundance. Hierarchical clustering was applied to both rows and columns to illustrate similarities among taxa, functions, and compartments.


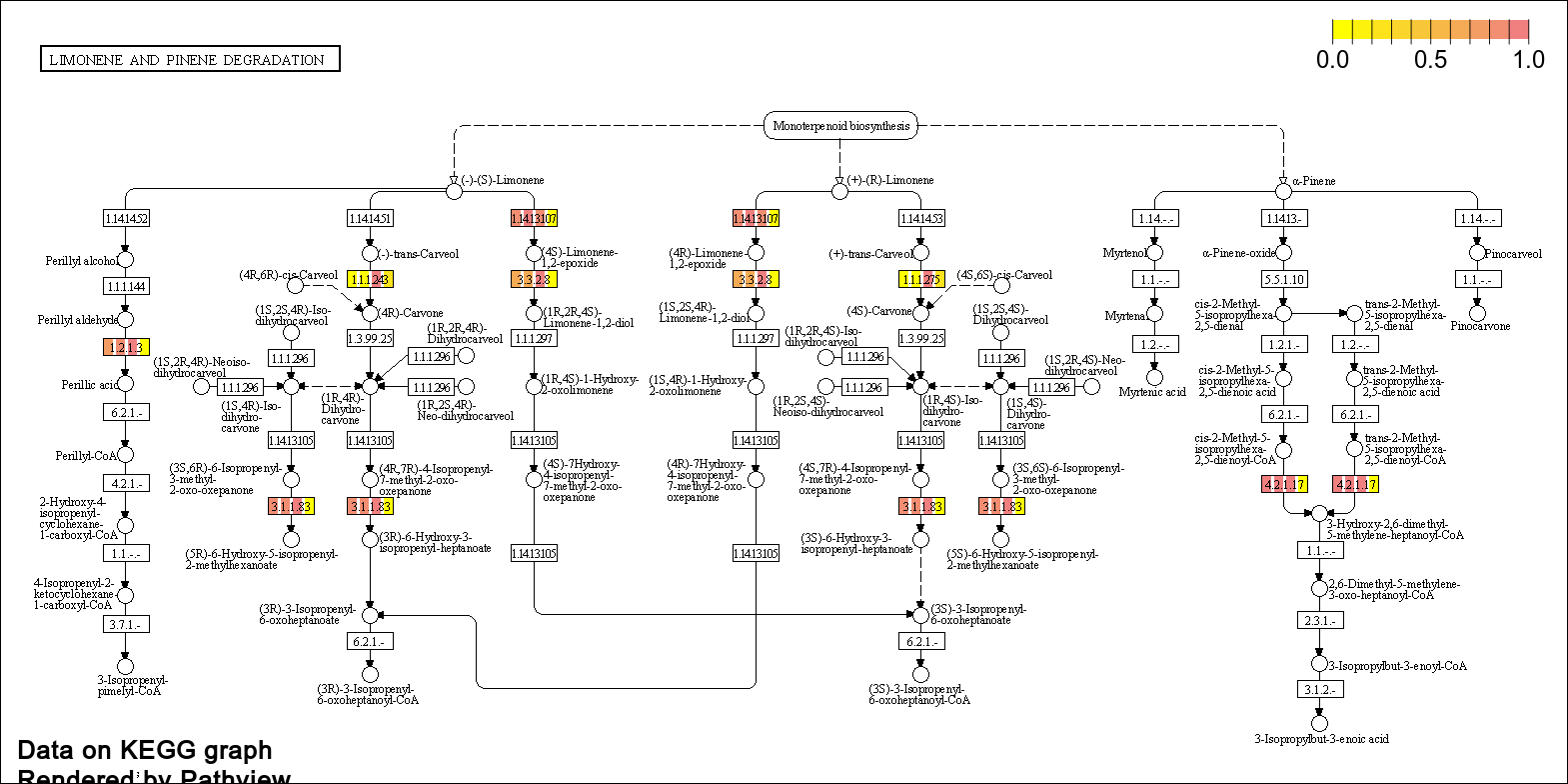


**Figure S4. KEGG pathway map of limonene and pinene degradation based on metagenomic functional annotation.** Heatmap coloring represents normalized gene abundance, with a gradient from low (light yellow) to high (dark red).
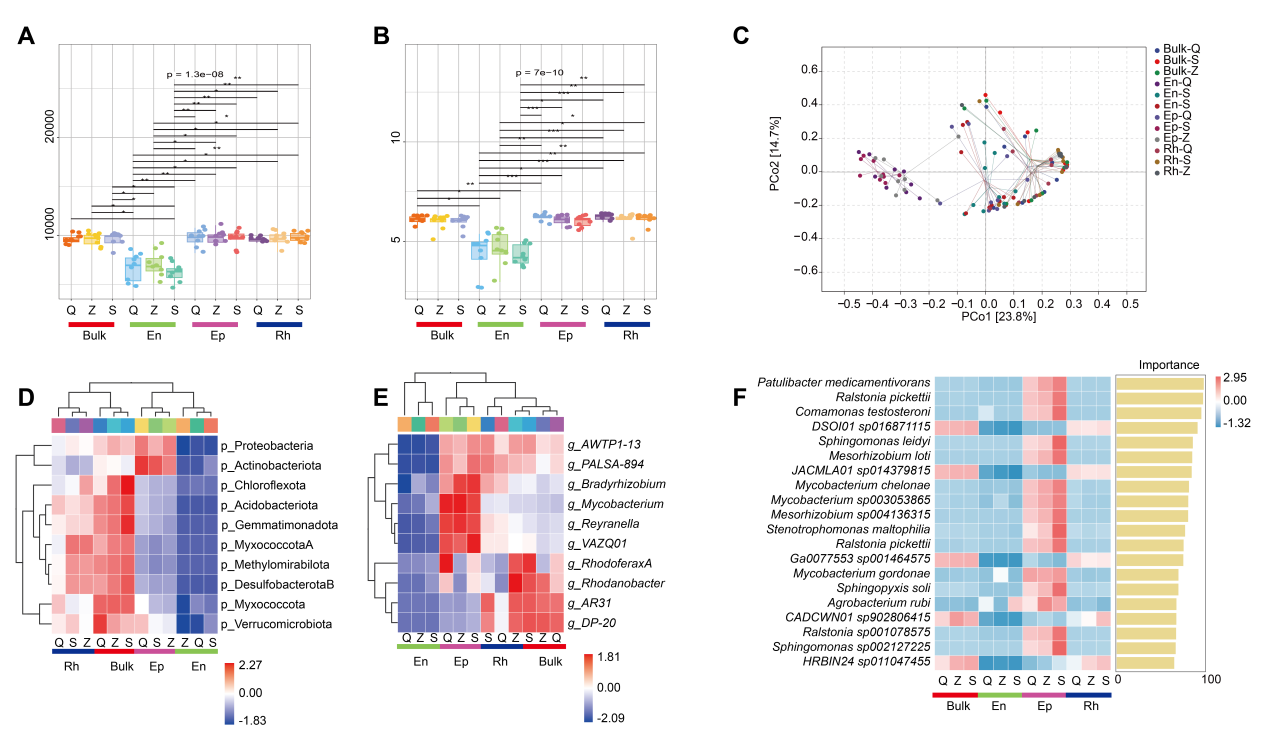


**Figure S5. Vertical and compartment-specific patterns of microbial diversity, community structure, and key discriminatory taxa.** (A) Chao1 richness index and (B) Shannon diversity index across three soil depths (Q, Z, S) within four compartments: bulk, rhizosphere (Rh), episphere (Ep), and endosphere (En). Significance levels are indicated. (C) Principal coordinates analysis (PCoA) based on Bray-Curtis distances showing beta-diversity separation among compartments and depths. (D) Heatmap of phylum-level relative abundances across compartments and vertical layers. (E) Heatmap of genus-level abundances highlighting compartment- and depth-specific taxa. (F) Random forest classification identifying microbial species with the highest importance in distinguishing compartments and depths.


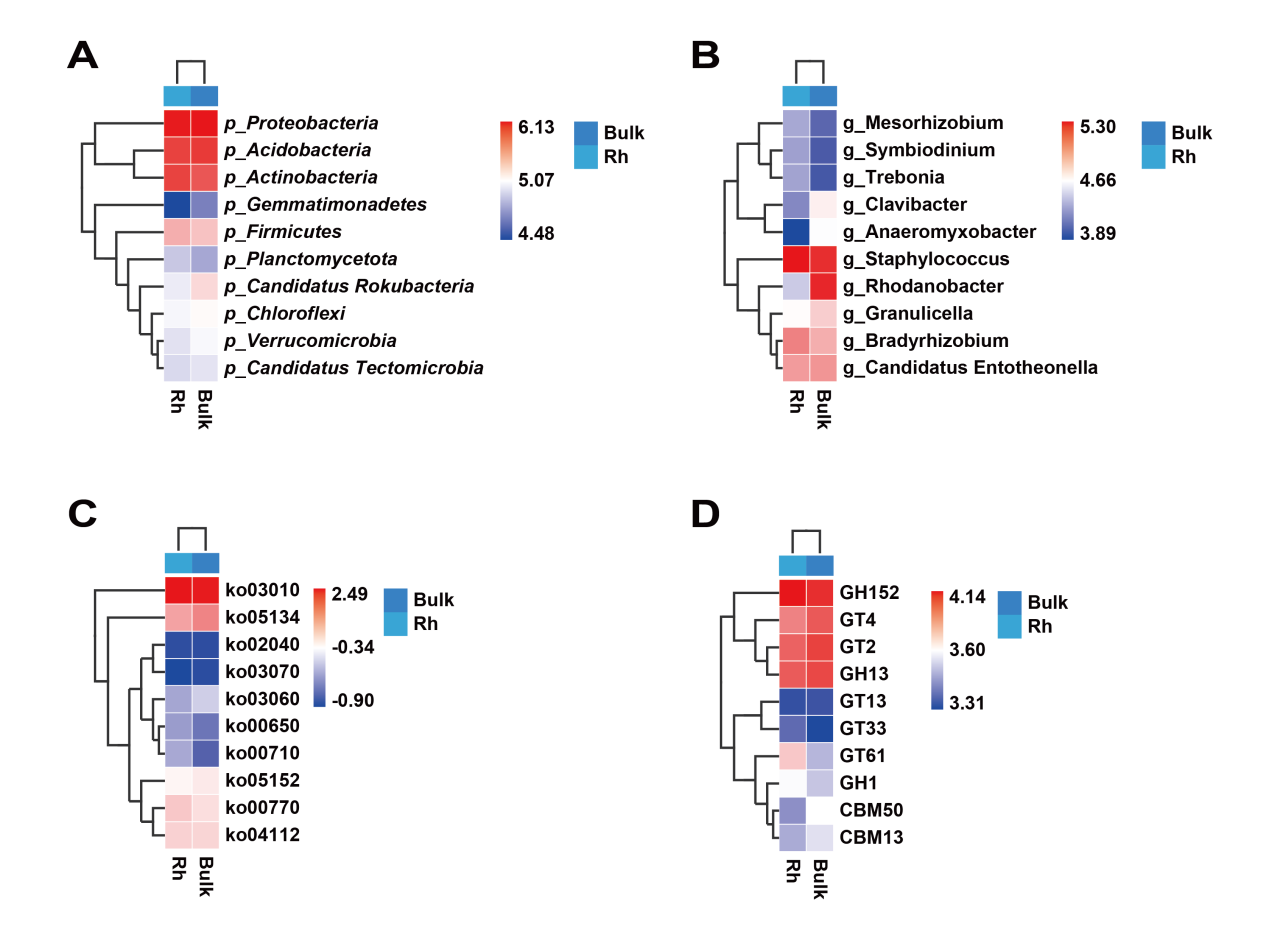


Figure S6. (A-B) Taxonomic composition of active microbial communities in non-rhizosphere and rhizosphere soils at the phylum (A) and genus (B) levels. (C) KEGG functional annotation of microbial transcripts. (D) Expression of carbohydrate-active enzyme (CAZyme) families. Heatmaps (A–D) are presented as Z-score–normalized expression values, with red representing higher expression and blue representing lower expression.


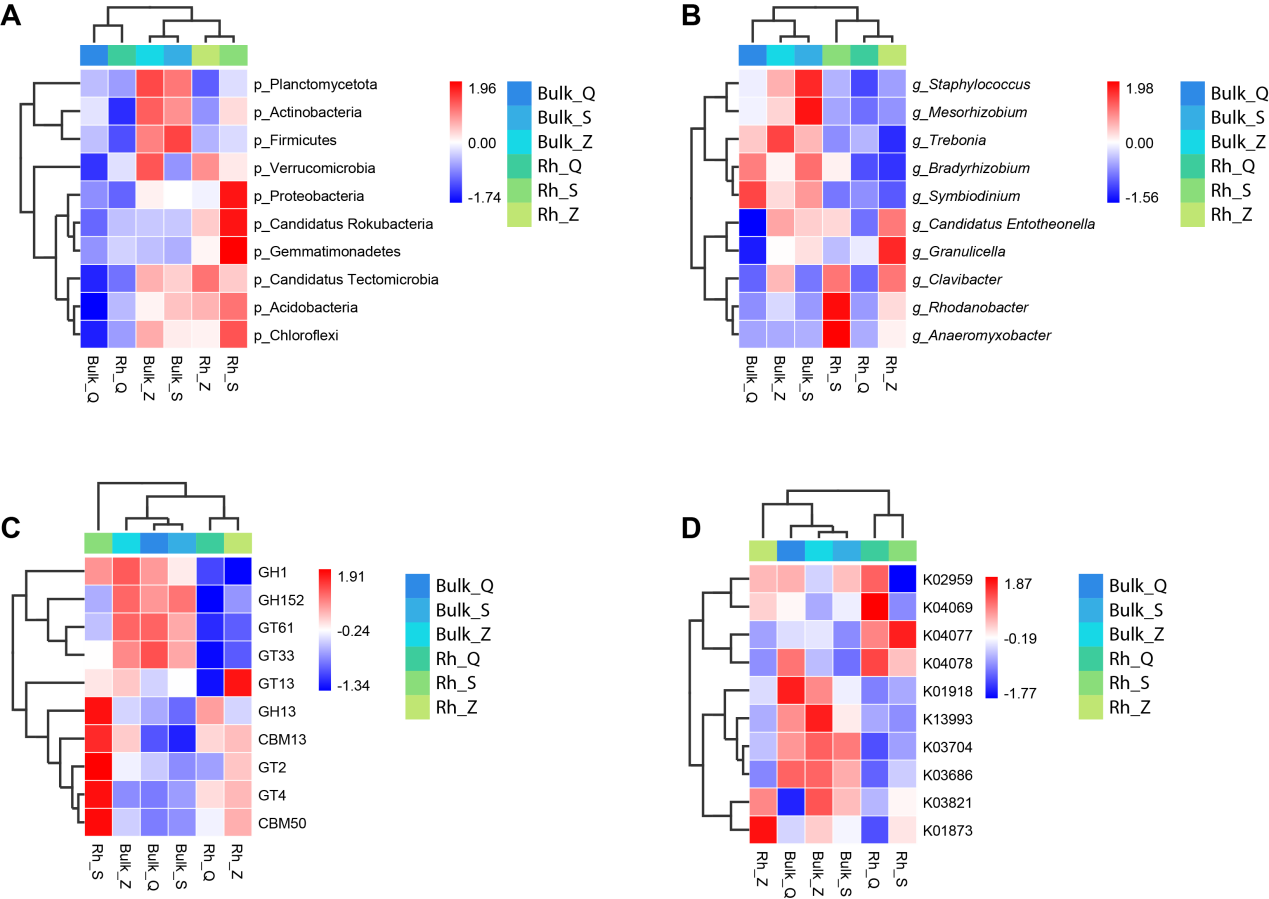


**Figure S7. Comparative heatmap analysis of microbial taxa, glycoside hydrolase families, and functional genes across treatments.** (A) Relative abundance of bacterial phyla. (B) Relative abundance of selected bacterial and algal genera. (C) Distribution of glycoside hydrolase (GH) and carbohydrate-binding module (CBM) families. (D) Abundance of functional genes (KEGG Orthologs, KOs).


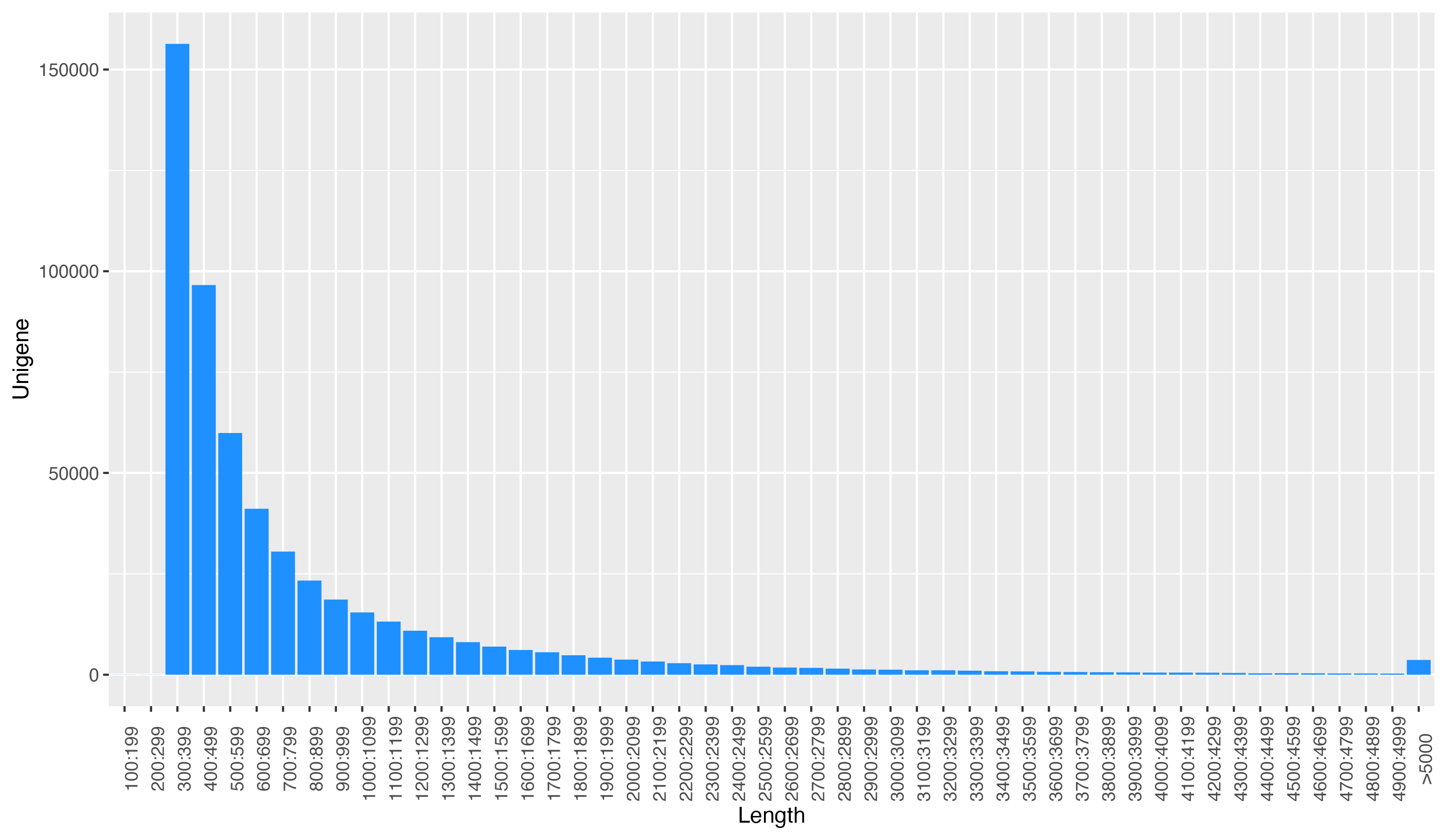


**Figure S8. Length distribution of unigenes obtained from root transcriptome sequencing of *Thuja sutchuenensis*.** The histogram shows the frequency of unigenes across different length intervals.


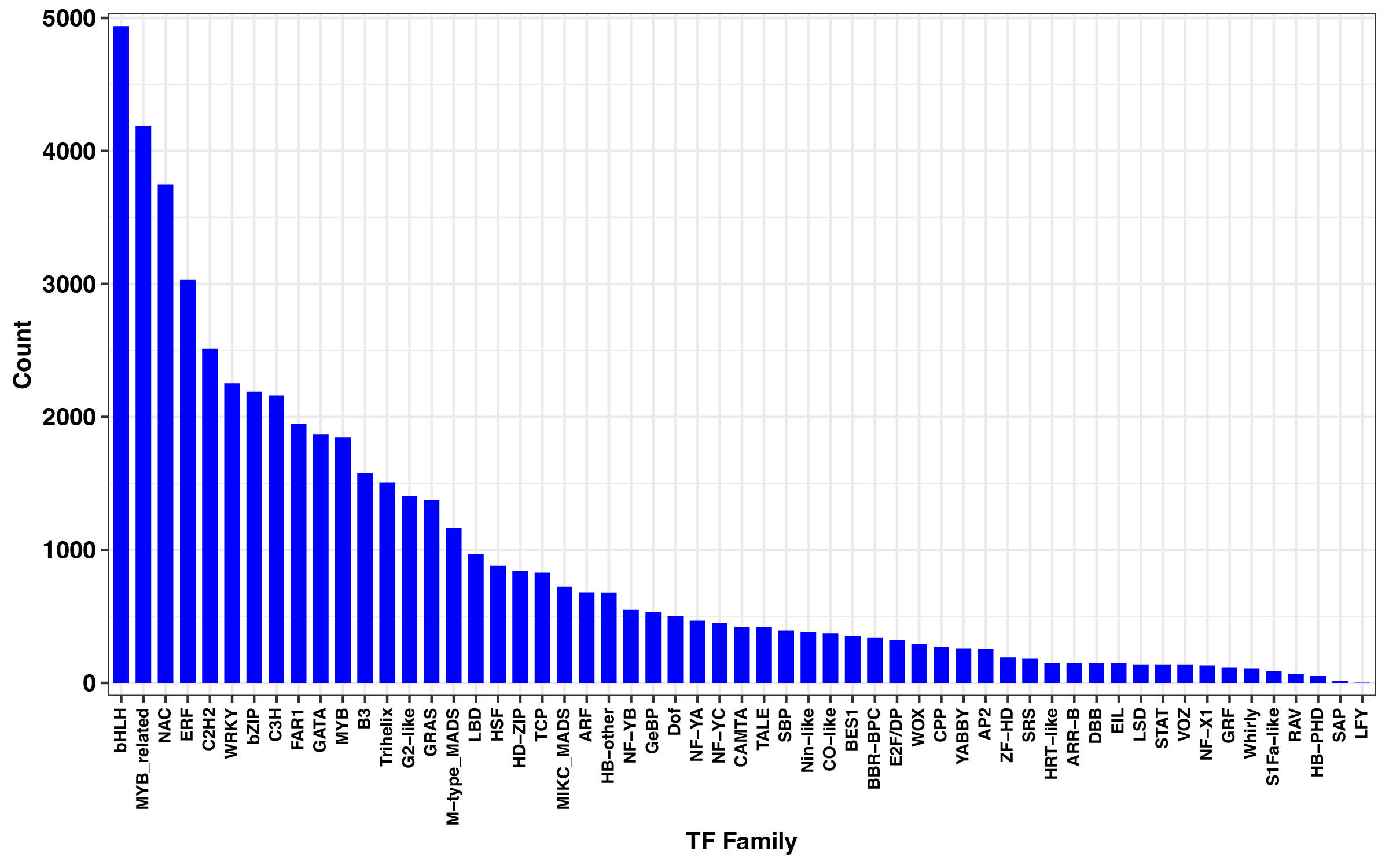


**Figure S9. Identification and classification of transcription factor (TF) families in the *Thuja sutchuenensis* root transcriptome.** The bar chart displays the number of unigenes associated with each transcription factor family. A total of 63 TF families were identified, with the most abundant being bHLH, MYB-related, NAC, ERF, and C2H2.


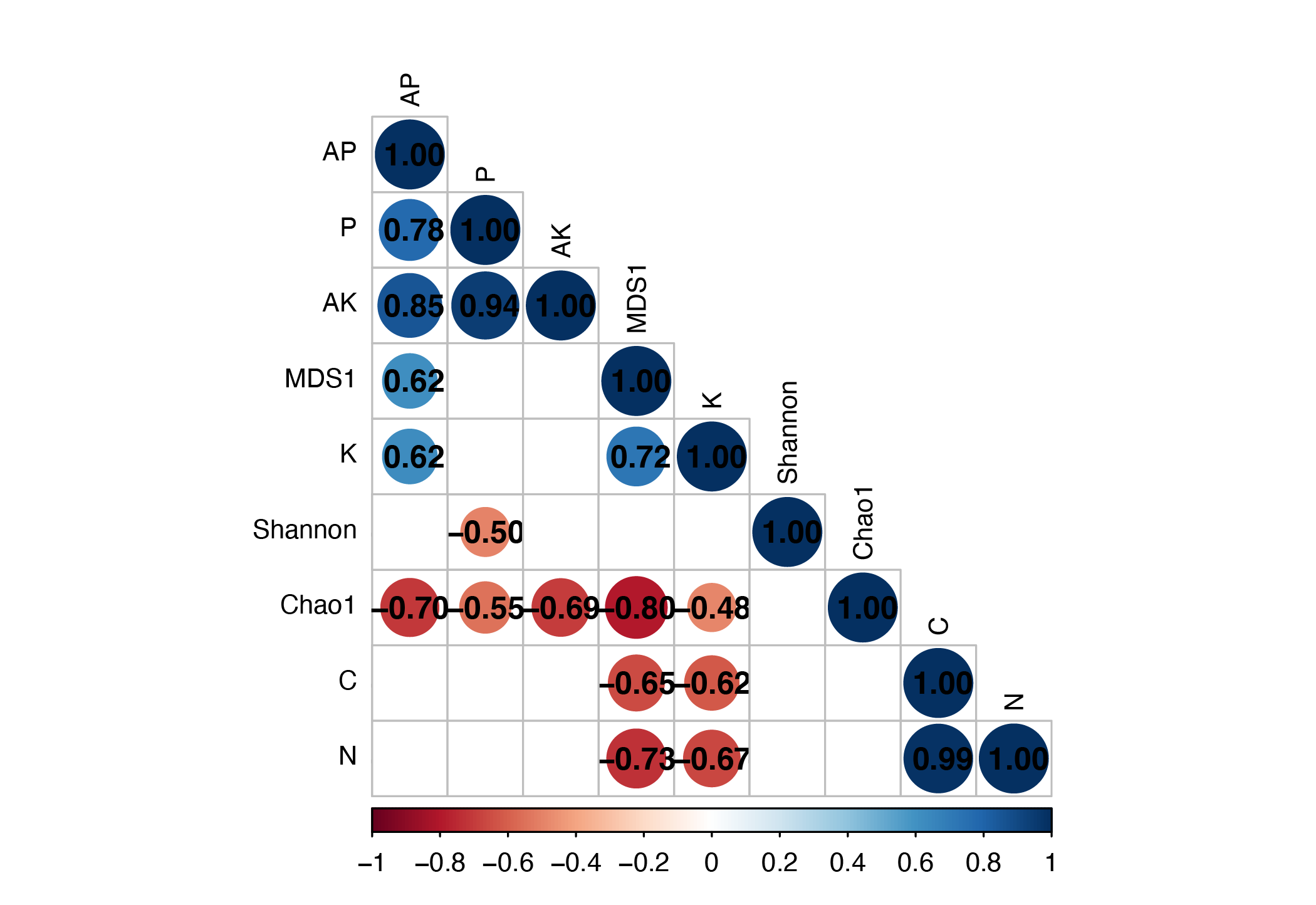


**Figure S10. Correlation analysis between soil physicochemical properties and microbial alpha and beta diversity metrics.** The heatmap displays Pearson correlation coefficients among soil properties, including total nitrogen (N), total carbon (C), phosphorus (P), available phosphorus (AP), potassium (K), available potassium (AK), and spatial beta diversity axis (MDS1), and microbial diversity indices (Chao1 richness and Shannon diversity). Circle size and color indicate the strength and direction of correlation (blue: positive; red: negative).

| **Table s1. Basic information of samping sites** | | | | | | |
| --- | --- | --- | --- | --- | --- | --- |
| Group | Longitude | Latitude | Elevation/m | Temperature | Humidity | Light intensity/lux |
| Site1 | 108.59651743°E | 31.63713276°N | 1211 | 30.5℃ | 75% | 9,697 |
| Site2 | 108.60728599°E | 31.6405262°N | 1378 |  |  |  |
| Site3 | 108.66182775°E | 31.56578969°N | 1362 |  |  |  |
| Site4 | 108.72866231°E | 31.65570226°N | 1185 |  |  |  |
| Site5 | 108.85664836°E | 31.59838064°N | 1468 |  |  |  |
| Site6 | 108.71327188°E | 31.65441371°N | 1410 | 29.5℃ | 74% | 10,140 |
| Site7 | 108.72350088°E | 31.6488939°N | 1385 |  |  |  |
| Site8 | 108.71329218°E | 31.65440427°N | 1409 |  |  |  |
| Site9 | 108.71454042°E | 31.65371351°N | 1414 |  |  |  |
| Site10 | 108.85664836°E | 31.59838064°N | 1468 |  |  |  |

**Table S2. Topological indexes of co-occurrence network**

| Index | Network |
| --- | --- |
| Degree | 284.8649 |
| Average_path_length | 1.6494 |
| Betweenness_centrality | 1739773.489 |
| Closeness_centrality | 6.7926 |
| Degree_assortativity | 0.5372 |
| Degree_centralization | 62440 |
| Density | 0.5405 |
| Cluster_num | 2 |
| Diameter | 5.4684 |
| Transitivity | 0.8735 |
| Num_vertice | 498 |
| Num_edge | 66886 |
| Modularity | 0.1385 |
